# Assessing validity of existing fistula-in-ano classifications in a cohort of 848 operated and MRI-assessed anal fistula patients – Cohort study

**DOI:** 10.1016/j.amsu.2020.09.022

**Published:** 2020-09-19

**Authors:** Pankaj Garg

**Affiliations:** a Garg Fistula Research Institute, Panchkula, India; b Indus International Hospital, Mohali, India

**Keywords:** Anal fistula, Classification, Parks, Fistulotomy, Sphincter

## Abstract

**Aim/background:**

The commonly used fistula-in-ano classifications, Park or St. James's University hospital(SJUH), neither grade fistulas as per their severity nor guide regarding their management. A new classification(NC), published in 2017, proposed to classify fistulas as per their severity and also guided in its management. The early grades (NC grade I & II) were simple fistulas and were amenable to fistulotomy whereas higher grades (NC grade III-V) were complex fistulas and were not amenable to fistulotomy.

**Methods:**

Lower grades of all the three classifications were classified as simple (Parks: I, SJUH:I-II, NC:I-II) whereas higher grades were classified as complex (Parks: II-IV, SJUH: III-V, NC: III-V) fistulas. Fistulotomy should be possible in simple fistulas but not in complex fistulas. This was analysed for all these classifications. The long-term follow-up of continence was done by an objective scoring system (Vaizey's scores).

**Results:**

The SJUH & Parks classifications categorized 504/828 fistulas as ‘complex’ which was quite inaccurate as 42.7%(215/504) of these fistulas were safely amenable to fistulotomy. On the other hand, the New classification (NC) classified 282/828 fistulas as ‘complex’ which was very accurate as 99% (279/282) of these were actually complex and were not amenable to fistulotomy. The change in the preoperative and the postoperative continence scores in the patients who underwent fistulotomy, as per these classifications, Parks & SJUH vs NC, was 0.064 ± 0.62 and 0.089 ± 0.85 respectively and was not significantly different(p = 0.80, Mann-Whitney *U* test).

**Conclusions:**

The New classification(NC) seems better than the existing classifications for grading the disease as well as in guiding the management of the disease.

## Highlights

• Largest MRI-based series of operated anal fistula patients to be ever published.
• Analysis of 848 fistula patients to assess the validity of existing classifications.
• Objective continence scores (Vaizey's scores) of each patient was evaluated.
• New classification (2017) was far more accurate than Parks and SJUH classifications.
• It accurately graded the fistulas as per their severity and guided their management.

## 1 Introduction

**Fig. 1:**
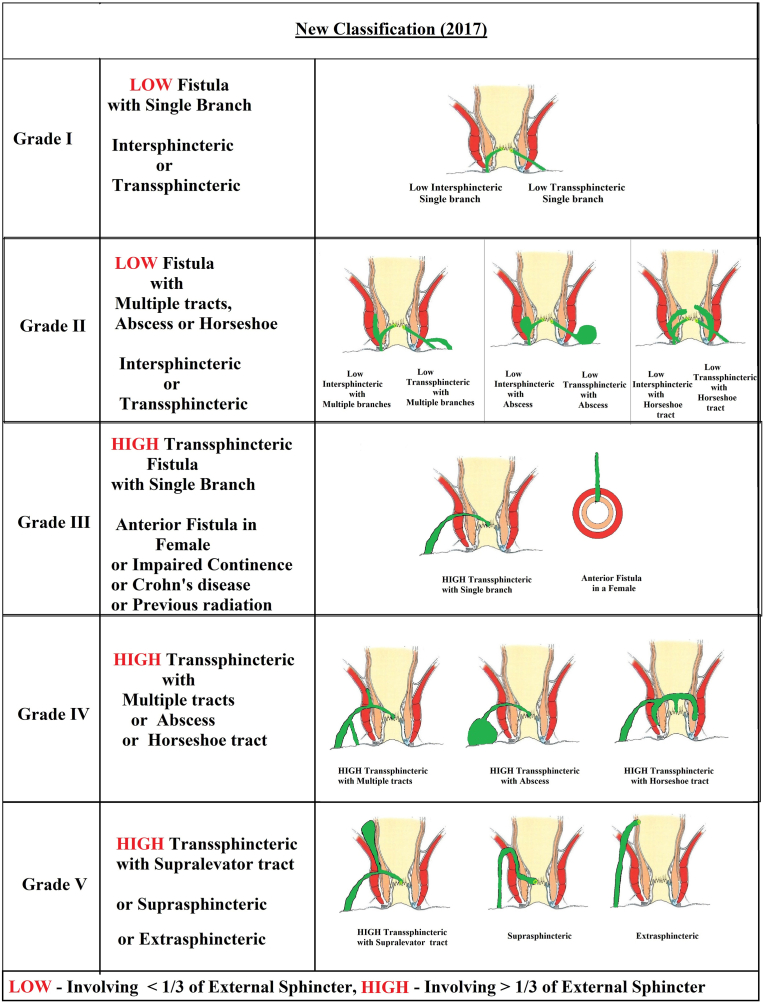
New classification (2017).

Fistula-in-ano is commonly classified as Parks [1], St. James's University hospital (SJUH) [2] or as a New classification, which was recently published (Table 1) (Fig. 1) [3,4].

**Table 1:** Existing classifications for anal fistula.

Classifications	Parks	St James's University Hospital (SJUH)	New classification (NC)
**Grade I**	- Intersphincteric	Linear Intersphincteric	- LOW linear intersphincteric or transsphincteric
**Grade II**	- Transsphincteric - Supralevator	Intersphincteric with abscess, multiple, or horseshoe tract	- LOW intersphincteric or transsphincteric with abscess, multiple, or horseshoe tract
**Grade III**	Suprasphincteric	Simple Transsphincteric	- High linear transsphincteric - Fistula with associated comorbidities#
**Grade IV**	Extrasphincteric	- Complex Transsphincteric - Suprasphincteric	-HIGH Transsphincteric fistula with abscess, multiple, or horseshoe tract
**Grade V**		- Supralevator - Extrasphincteric	- Supralevator - Suprasphincteric - Extrasphincteric

Parks- Supralevator fistula could be in grade I, II or III.

SJUH- Suprasphincteric fistula was categorized in grade IV.

LOW Fistula- < 1/3 of external sphincter involvement, HIGH Fistula->1/3 sphincter involvement.

# Crohn's disease, sphincter injury, post radiation exposure or anterior fistula in a female.

The purpose of any classification is that it should guide regarding the.

1. Severity of the disease
2. Management of the disease: The lower grade disease is expected to be managed easily. As fistulotomy is the commonest, simplest, easily reproducible and most widespread procedure for fistula-in-ano [5], lower grades should be safely amenable to fistulotomy and higher grade (complex fistula) should not be amenable to fistulotomy.

In the Parks classification, all intersphincteric fistulas were categorized as grade I whereas all transsphincteric fistulas were classified as grade II (Table 1). Though the SJUH classification was MRI based, it was essentially same as the Parks classification (Table 1). SJUH simply bifurcated first two grades of Parks into four grades (Table 1). So practically both these classifications were similar and didn't help either in grading the fistula according to its severity or guiding in the disease management.

The New classification (NC), published in 2017, supposedly graded the fistula as per its severity and also guided in its management [4]. NC recommended that grade I-II were simple fistulas and could be safely managed by fistulotomy without any risk to the continence. On the other hand, NC grade III-V were complex fistulas and fistulotomy should not be attempted in these fistulas as that would increase the risk of incontinence [[3], [4], [5]]. It was recommended that the grade III-V fistulas should only be managed with one of the sphincter-saving procedures.

## 2 Methods

All consecutive patients suffering from anal fistula operated at a single centre over a period of seven years from January 2013 to January 2020 were included in the cohort study.

The surgery was primarily decided on basis of the amount of involvement of the external sphincter. Preoperative evaluation with MRI was done in all the patients. The amount of the external-sphincter involvement was assessed meticulously on the clinical examination, examination under anesthesia and through MRI scan before proceeding with the surgery. Fistulotomy was done in low fistulas (<1/3 external-sphincter involvement) and a sphincter-sparing procedure was done in high fistulas (>1/3 external-sphincter involvement). This was done to ensure that no patient with high fistula should undergo sphincter cutting procedure. This protocol was chosen to select the operative procedure. The categorization of fistulas in three different classifications was done retrospectively.

The patients were discharged from the hospital on the day after surgery. They were regularly followed-up at clinic for 1–2 weeks, as required. After that, the patients were monitored daily by online communication (WhatsApp) and a monthly physical visit to the institute.

Their continence was evaluated by Vaizey's continence scoring [6] preoperatively as well as postoperatively (at 3 months and on long-term follow-up). This scoring assessed six parameters - incontinence to gas, liquid or solid, any need to take constipation medicines, any alteration in lifestyle, inability to hold defecation for 15 min and need to wear a pad. A score of 24 would mean that the patient was totally incontinent while a score of zero would imply perfect continence [6]. Vaizey's scores were used as it is more comprehensive than other scoring systems.

In the study, fistulas of all the patients were categorized as per all the three classifications- Parks, SJUH and New classification (NC). Lower grades of each classification were classified as ‘simple’ (Parks grade I, SJUH grade I-II, NC grade I-II) whereas higher grades were classified as ‘complex’ (Parks grade II-IV, SJUH grade III-V, NC grade III-V) fistulas (Table 1). Amenability of the fistulas to fistulotomy was analysed as per all the classifications. The data of 440 patients out of the present cohort had been previously published [3]. That was the paper in which the New Classification was proposed [3], whereas this manuscript, apart from having a significant rise in the number of subjects, also attempts to ascertain the validity of the 3 classifications by analysing the long-term continence scores. The approval for this was taken from the Institute Ethics Committee of the hospital via reference no. Indus hospital/EC/04–12. The study was registered at researchregistry.com via unique identifying number (UIN)- researchregistry2239 [7]. The work has been reported in line with the STROCSS criteria [8].

## 3 Statistical analysis

The categorical variables were compared by performing Fisher's exact test or chi-squared analysis. In a normally distributed data, the continuous variables were analysed by *t*-test, when there were two samples, or ANOVA test, when there were more than two samples. In case the data was not distributed normally, Wilcoxon Signed Rank test was done for the paired samples and Mann-Whitney *U* test was performed for the unpaired samples. The significant cut off point was set at p < 0.05.

## 4 Results

In a retrospective study, the utility of the existing classifications was analysed in 848 fistula-in-ano patients, operated at a specialized fistula centre between January 2013 to January 2020. The follow-up ranged from 6 to 84 months (median-36 months).

The SJUH & the Parks classifications categorized same number of patients in simple (344) and complex (504) category. They classified 344/828 in the ‘simple’ category which was accurate, as almost all of them (308/344) could undergo fistulotomy safely, with no significant change in the continence scores after the surgery (mean pre-operative vs postoperative scores were 0.029 ± 0.39 & 0.112 ± 0.57 respectively, p = 0.45, not significant) (Table-2 & 3). However, these two classifications classified 504/828 fistulas in the ‘complex’ category which was quite inaccurate as 42.7% (215/504) of these fistulas categorized as ‘complex’ were safely amenable to fistulotomy (Table 2).

**Table 2:** Amenability of fistulas to fistulotomy as per existing classifications.

Classification	Grade	Total (n = 848)	Amenable to fistulotomy (n = 523)	Comment
**Parks**	Simple = I	344	308 (89.5%)	42.7% of fistulas classified as ‘complex’ were amenable to fistulotomy. This is a major flaw.
	Complex = II + III + IV	504	215 (42.7%)	
**SJUH**	Simple = I + II	344	308 (89.5%)	42.7% of fistulas classified as ‘complex’ were amenable to fistulotomy. This is a major flaw
	Complex = III + IV + V	504	215 (42.7%)	
**New classification (NC)**	Simple = I + II	566	520 (91.9%)	Only 1% of fistulas classified as ‘complex’ were amenable to fistulotomy. These 3 were anterior fistula in females and fistulotomy could be done safely.
	Complex = III + IV + V	282	3 (1.0%)	

On the other hand, the New classification (NC) classified 566/828 patients in the ‘simple’ category. This was accurate as 520/566 patients could undergo fistulotomy safely with no significant change in continence scores after the operation (mean pre-operative vs postoperative scores were 0.044 ± 0.52& 0.135 ± 0.67 respectively, p = 0.14, not significant) (Table 2, Table 3). NC classified 282/828 fistulas in ‘complex’ category which was also very accurate as 99% (279/282) of these were complex and were not amenable to fistulotomy (Table 2).

**Table 3:** Change in Objective Continence Scores in patients who underwent fistulotomy.

Patients who were classified as ‘simple’ and underwent Fistulotomy (n = 523)	Pre-Operative Scores (Mean)	Post-Operative Score (Mean)	Mann-Whitney *U* test	Change in Continence [Postoperative Scores – Preoperative scores] (Mean)	Mann-Whitney *U* test
As per **Parks & SJUH** classifications (**n** = **308**)	0.029 ± 0.39	0.112 ± 0.57	(p = 0.45)^a^	0.064 ± 0.62	**P** = **0.80**^**c**^
As per **New classification (NC)** (**n** = **520**)	0.044 ± 0.52	0.135 ± 0.67	(p = 0.14)^b^	0.089 ± 0.85	

a = p value of comparison of the preoperative and the postoperative continence scores after fistulotomy in fistulas classified as ‘simple’ as per the Parks & the SJUH classifications.

b = p value of comparison of the preoperative and the postoperative continence scores after fistulotomy in fistulas classified as ‘simple’ as per the New classification (NC).

c = p value of comparison of change in continence scores (postoperative - postoperative continence scores) after fistulotomy in fistulas classified as ‘simple’ as per Parks & SJUH classification Vs NC.

The difference between preoperative and postoperative objective continence scores in all the patients who should have underwent fistulotomy, as per each classification, was tabulated and compared (Table 3). As per Parks and SJUH classification, 308 patients underwent fistulotomy and the change in continence scores after the operation was 0.064 ± 0.62. On the other hand, as per NC, 520 patients underwent fistulotomy and the change in continence scores after the operation was 0.089 ± 0.85. This change in preoperative and postoperative continence scores, between Parks & SJUH vs NC, was not significantly different (p = 0.80, not significant, Mann-Whitney *U* test) (Table 3).

## 5 Discussion

The results of this study highlighted that 42.7% (215/504) of the fistulas, classified as ‘complex’ by the SJUH & the Parks classifications, were actually ‘simple’ as fistulotomy could be done in these patients without any loss of continence. Thus, these two classifications tend to categorize much more ‘simple fistulas’ (safely amenable to fistulotomy) into the ‘complex’ category. This is a major lacuna. The disadvantage was that these ‘simple’ fistulas, erroneously categorized as ‘complex’ (42%) by the SJUH & the Parks classifications, could have been conveniently and safely managed by fistulotomy, which has a high success rate (90–98%). But if the management of these fistulas was done like ‘complex’ fistulas, then they would have ended up in undergoing one of the sphincter-saving procedures. These sphincter-saving procedures have a lower success rate (40–70%) than fistulotomy [9,10], thereby unnecessarily increasing the risk of recurrence of the fistulas, increasing morbidity and suffering.

The fistulas which were categorized as ‘simple’, and subsequently underwent fistulotomy, were much more according to NC (n = 520) than those according to Parks & SJUH (n = 308). In order to ascertain the validity of NC, the change in continence scores after fistulotomy (postoperative – preoperative continence scores) were compared between Parks & SJUH (n = 308) and NC (n = 520). In case NC erroneously categorized ‘complex’ fistulas in the ‘simple’ category, and subjected them to fistulotomy, then the continence levels would deteriorate much more in the NC group. The analysis showed that the change in preoperative and postoperative continence scores, between the Parks & SJUH vs NC, was 0.064 ± 0.62 and 0.089 ± 0.85 respectively and was not significantly different (p = 0.80, Mann-Whitney *U* test, not significant) (Table 3). This highlighted that the deterioration in continence in the patients undergoing fistulotomy, in the NC group, was comparable to the Parks and SJUH group. Thus, these results confirmed that NC was accurate in classifying higher number of patients in the ‘simple’ category.

8–10% of patients, who were categorized as ‘simple’ fistula by each classification, and could have safely been managed by fistulotomy, opted for sphincter-sparing procedure even after knowing that they had simple fistula. (Table 2). Those were their personal preferences (avoiding fistulotomy even in low fistulas) and had to be respected.

These results corroborated that NC could accurately guide regarding the disease management (NC grade:I, II were safely amenable to fistulotomy whereas NC grade:III–V should undergo a sphincter-sparing procedure). Park and SJUH were quite inaccurate in guiding about the management, as they categorized a greater number of patients in the complex category. These two classifications were also inaccurate in grading the disease as per its severity. For example, a very low transsphincteric fistula, involving only 10% of the external sphincteric with a small abscess, would be categorized as ‘complex’ by these classifications (Parks-II, SJUH-IV) but would be classified as ‘simple’ by NC (grade II). Such a fistula can easily be managed by fistulotomy.

Apart from the two basic flaws in the Parks and the SJUH classifications (they didn't grade fistulas as per their severity and didn't guide regarding the management of the disease), there were other lacunae in these classifications as well. First, Parks classification was based on the experience of 400 fistula patients, but the diagnosis of these fistulas was not validated/corroborated by MRI/TRUS [1]. Second, the status of the supralevator fistulas was not clearly stated in the Parks classification [1]. It could occur in any of the Parks grades I, II or III(Table 1) [1]. Third, a full grade (grade IV) was assigned to the extrasphincteric fistulas (Table 1). There is increasing evidence that the extrasphincteric fistulas either do not exist or are extremely rare [11]. Fourth, no category was assigned to the patients with comorbidities, like anal fistulas with Crohn's disease, anterior fistula in a female, previous irradiation, weakened sphincter due to previous operations, etc.

Similarly, the SJUH classification had several additional flaws. In SJUH, the suprasphincteric fistulas were categorized along with the transsphincteric fistulas (Table 1) [2]. This is a major error as the management of the suprasphincteric fistulas is much more difficult than the transsphincteric fistulas, and the prognosis is also much worse than the transsphincteric fistulas. This classification was not validated by any patient data [2]. Despite utilizing the MRI scans and the increment of knowledge of a quarter century (Parks classification was published in 1976 and SJUH was published in 2000) [1,2], this classification failed to improve over the Parks classification. Functionally, SJUH was almost same as the Parks classification [4]. That is why it failed to replace the Parks classification. Again, as in the Parks classification, the patients with comorbidities were not included in the classification (Table 1).

The New classification (NC) has attempted to remove the shortcomings in the Parks and the SJUH classifications (Fig. 1). This was the first classification to grade fistulas as low or high fistulas, based on the ‘extent of involvement of the external sphincter’, rather than as intersphincteric or transsphincteric fistula. This was also the first classification which guided the surgeons regarding the management of the disease. It also graded fistulas according to the severity of the disease. Unlike previous classifications, there was clarity on suprasphincteric, supralevator and extrasphincteric fistulas, and they were clubbed together as grade V fistulas (Fig. 1). In NC, patients with comorbidities were categorized as grade III fistulas. It was validated by a large MRI-based series of 440 operated fistula patients [3]. Now, the present study, which has the largest MRI-based series of operated fistula-in-ano patients (n=848), has further corroborated the accuracy of this classification.

The study had few limitations. First, the study was retrospective. Second, it would have been better if the long-term continence could have been evaluated by anal manometry.

To conclude, the New classification (NC) is an improvement and is better than the existing classifications for grading the disease and in guiding about the management. Therefore, NC is recommended to be used by the radiologists, to guide general surgeons as which fistulas can be managed easily by fistulotomy (grade I-II) and which should be referred to an expert fistula surgeon (grade III-V). Further studies are needed to corroborate the findings of the study.

## Declaration of competing interest

Author, Dr Pankaj Garg declares that they have no conflict of interest.
